# From spontaneous rhythmic engagement to joint drumming: A gradual development of flexible coordination at approximately 24 months of age

**DOI:** 10.3389/fpsyg.2022.907834

**Published:** 2022-09-29

**Authors:** Lira Yu, Kaho Todoriki, Masako Myowa

**Affiliations:** ^1^Graduate School of Arts and Sciences, The University of Tokyo, Meguro, Japan; ^2^Graduate School of Education, Kyoto University, Kyoto, Japan

**Keywords:** joint drumming, spontaneous motor tempo, tempo adjustment, synchronization, entrainment, inhibitory control, action prediction

## Abstract

Humans have a flexible and accurate ability to coordinate their movement in time with external rhythms. However, it remains unclear when and how, during their development, human children acquire the ability to adjust tempo and control the timing of their movement toward others. A previous study suggested that such self-regulation of coordination develops at around 18 and 30 months after birth. In this study, we investigated the performance of 24-month-old children and compared their data with those of 18- and 30-month-olds provided in our previous study. In the joint-drumming task, each child was enticed to drum under four conditions [partner: mother or robot; speed: 400 or 600 ms inter-stimulus-interval (ISI)]. The most pivotal test condition was the 600 ms ISI speed condition (slower than children’s spontaneous motor tempo in these age groups). We found that from the age of 24 months, children try to slow down their drumming tempo toward the 600 ms ISI speed condition, regardless of the drumming partner. On the other hand, significant timing control toward the onset of the 600 ms ISI condition was observed from the age of 30 months. This implies that both motor and cognitive mechanisms are required for flexible tempo adjustment and accurate synchronization and that these develop gradually among 18-, 24-, and 30-month-olds.

## Introduction

Humans have a flexible and accurate ability to coordinate their movement to match external rhythms. For example, while listening to music, we often produce finger/foot-taps to the music beat. When walking with another person, we tend to match our stride with others without attentive effort. Such an ability to coordinate one’s movement in time with external rhythms is known to be one of the most important social-cognitive abilities in humans, since it enables us to establish smooth social interaction, communication, and cooperation with others (McNeill, 1997).

When and how do infants coordinate their movements in time with their external rhythms? In infants under 2 years of age, spontaneous rhythmic engagement with music, also referred to as *entrainment*, has been observed in a musical context (e.g., Zentner and Eerola, 2010; Fujii et al., 2014; Rocha and Mareschal, 2017; Cirelli and Trehub, 2019). The infants in these studies mostly demonstrated bouts of repetitive movements using the limbs, torso, or head and some extent of tempo flexibility. Conversely, in children over 2 years of age, studies that investigated specific target movements, such as tapping or drumming behaviors, demonstrated that children begin to show persistent rhythmic movements and flexible and accurate coordination with auditory rhythms (e.g., Drake et al., 2000; Provasi and Bobin-Bègue, 2003) or to a drumming partner (e.g., Kirschner and Tomasello, 2009; Endedijk et al., 2015). These studies suggest that flexible tempo adjustment and accurate synchronization appear in children as young as 2.5 years. However, since there are few studies examining children that are both under and above 2 years in the same experimental conditions (but see Kragness et al., 2022), it is still unclear whether, and to what extent, children under 2 years have coordination abilities compared to older age groups.

Our previous study extended the testable minimum age by adapting a joint-drumming task from Kirschner and Tomasello (2009) as follows: (1) introducing a drumstick to be used for drumming, (2) setting the participants’ mother as a social partner, and (3) playing a well-known song (“Twinkle Twinkle Little Star”) during the test phase to elicit the participants’ repetitive drumming (see Yu and Myowa, 2021 for more detail). Those adaptations enabled us to examine 18-, 30-, and 42-month-old children’s abilities under the same experimental conditions. The results showed that tempo adjustment toward the 400 ms ISI condition, which is close to the spontaneous motor tempo (SMT) for these age groups (Provasi and Bobin-Bègue, 2003; Bobin-Bègue and Provasi, 2008; Rocha et al., 2021), was observed in children as young as 18 months old. In contrast, tempo adjustment and synchronization ability toward the 600 ms ISI condition were observed from the age of 30 months, regardless of the drumming partner (i.e., mother or robot). A subject’s SMT slows with age during childhood, and as the SMT in adults is approximately 600 ms (Fraisse, 1982; McAuley et al., 2006), movement coordination under the 600 ms ISI condition may be difficult for children. Overall, the findings suggest that flexible tempo adjustment and accurate synchronization develops between the ages of 18 and 30 months.

Flexible coordination is also necessary for turn-taking. Meyer et al. (2015) assessed the ability to predict others’ actions (action prediction) and control one’s own actions (inhibitory action control) in 30-month-old children in a turn-taking game. They demonstrated that action prediction was related to turn-timing variability (i.e., time interval between the last button press by the adult experimenter and the button press of the child), whereas inhibitory action control was related to turn-taking accuracy (i.e., a correct execution of button pressing in alternation between the adult partner and the child). This suggests that both motor and cognitive abilities play distinct roles in early joint action coordination.

Based on these findings, the current study aimed to reveal when children develop the ability of flexible tempo adjustment, as well as how motor development and cognitive ability to predict others’ actions affect development. We examined 24-month-olds under the joint-drumming task and compared their data with those of 18- and 30-month-olds from our previous study (Yu and Myowa, 2021). Regarding flexible tempo adjustment, we examined whether children’s drumming tempo significantly changed depending on the speed condition (400 or 600 ms ISI condition). For the children’s motor development, we examined their SMT from their free-drumming responses during the familiarization phase. Regarding the ability to predict others’ actions, we examined the children’s synchronization ability by performing Rayleigh’s test. We hypothesized the following: 1) a tempo adjustment toward a slower tempo than the children’s SMT will develop gradually in 18-, 24-, and 30-month-olds; 2) the ability to perform a flexible tempo adjustment will require a slowdown of the SMT; and 3) the ability to perform a flexible tempo adjustment will require accurate synchronization.

## Materials and methods

### Participants

Nineteen 24-month-old children participated in the experiment (12 girls and 7 boys; mean age = 24.21 months; range = 23.54 to 25.02 months). Three additional children were excluded from the analysis because of excessive fussiness. The children’s parents were asked to provide written informed consent before participation. The study protocol adhered to the Declaration of Helsinki and was approved by the ethics review board of the Kyoto University Unit for the Advanced Study of the Mind. Data for 18-month-olds (10 girls and 8 boys; mean age = 18.21 months; range = 17.52 to 18.74 months) and 30-month-olds (9 girls and 9 boys; mean age = 30.17 months; range = 28.60 to 31.89 months) were taken from our previous study (Yu and Myowa, 2021).

### Apparatus and stimuli

We used the same apparatus and stimuli as in our previous study (Yu and Myowa, 2021). For the drumming behavior, two sets of toy drums (24.5 cm diameter, 12 cm high) and toy drumsticks (15 cm length) were used. A vibration sensor (Piezo film sensor) was attached beneath each drumming surface and a PC data logger (U3HV-LJ, LabJack Co.) was used to record the signals from both drums. A preprogrammed drumming robot was introduced to examine the partner’s effect (i.e., mother or robot). A digital video camera (HDR-CX670, SONY) was used to film the participants and their mothers throughout the experiment. For each trial, a speaker (Fostex PM0.1) played background music, namely the melody of “Twinkle Twinkle Little Star” and Audacity 2.4.2 was used to create versions of the two speed conditions (i.e., 400 and 600 ms ISI conditions). Sixty-four beats were presented regardless of the speed condition. Thus, the durations of the 400 and 600 ms ISI conditions were 25 s and 38 s, respectively.

### Procedure

This study was conducted with the cooperation of the participants’ mothers. After receiving informed consent, the experimenter (author LY) explained further procedures to the mother, including the verbal instructions to be given to the child, depending on the condition. A practice for matching drumming in time with the presented music was also conducted. During this time, a research assistant (author KT) interacted with the child with a few toys and books, beside the parent and experimenter.

**Figure 1:**
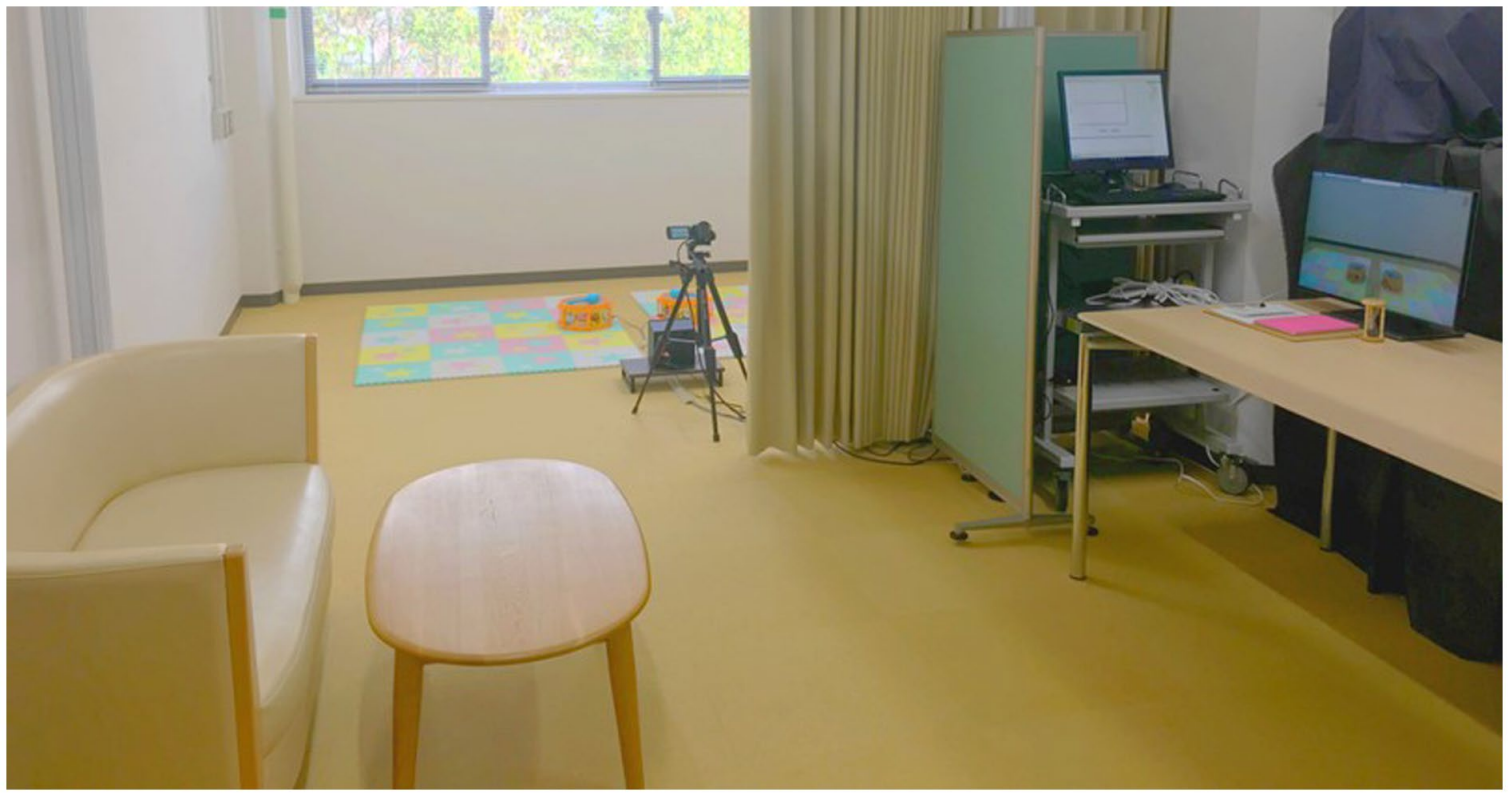
Waiting area (front) and experimental space (back) of this study.

Following a brief warm-up period in the waiting area that contained the PC and monitors used in the study, the child and mother moved to the experimental space with the experimenter. The experimental space was separated from the waiting area using a curtain (Figure 1). The child and mother sat next to each other on a floor mat, and the experimenter sat in front of them on another floor mat. After the experimenter placed a drum on each floor mat and handed one drumstick to the child, a familiarization phase consisting of three sequences started as follows: (1) the child was encouraged to drum freely while the experimenter sat in front of the child; (2) the child and mother were allowed to drum freely while facing each other, each using one drum; and (3) the child was introduced to the drumming robot, named *Shikaku-chan*, and was allowed to drum freely with the robot or touch it. Each situation lasted no longer than 1 min to avoid the child’s loss of interest in drumming. No music was played throughout the familiarization phase.

Following the familiarization phase, the experimenter left the experimental space, drew the curtain, remained in the waiting area, and started the test phase. Each participant was tested under four conditions in a single day (partner: mother or robot; speed: 400 or 600 ms ISI). The order of speed and partner under the same speed condition was counterbalanced across participants. Each condition was repeated only once. The second trial was conducted only when the child did not drum at all in the first trial. Each trial started with verbal instructions provided by the child’s mother. In the mother condition, the mother said, “Shall we play the drum together now?” while sitting in front of the child. A few seconds later, the melody of “Twinkle Twinkle Little Star” was played, and the mother began drumming along with every beat of the music. In the robot condition, the mother said, “Can you drum with *Shikaku-chan* now?” while sitting beside the robot. A few seconds later, the robot began drumming and music was played to accompany the movement of the robot. In all conditions, the mother unconditionally praised the child after the trial ended. Between the conditions, the children were given stickers to play with for approximately 3 min.

### Data analysis

To assess the children’s ability to make tempo adjustments, the median of inter-response-intervals (IRIs) was calculated for each trial. To test whether children changed the drumming tempo depending on the speed condition (400 or 600 ms ISI), the Kruskal–Wallis rank sum test was conducted for each age group in both mother and robot conditions. We calculated the median IRIs only when the trial included more than four repeated drumming hits (i.e., more than three IRIs), without unusually large IRIs. If we detected an unusually large IRI that exceeded 2 s, we checked the videos. We eliminated large IRIs if the child changed the drumming hand, took a break (i.e., hands free from drumming), or drummed without hitting the drum surface. As in our previous study, we checked the mothers’ drumming tempo for the mother condition. In the 24-month-old group, no data were excluded due to the mother’s too fast or too slow drumming tempo (±5% from the designated speed).

To measure the children’s SMT, the free-drumming responses during the familiarization phase were examined. The SMT of the 24-month-old group was extracted from the responses observed when the children drummed alone in front of the experimenter.

To assess the children’s ability to synchronize, Rayleigh’s test was performed for each trial. This tests the null hypothesis of circular uniformity. Rejection of the null hypothesis indicated that the children controlled their drumming at a specific timing in response to the onset of their partner’s drumming. We performed Rayleigh’s test only when the trial included more than 10 drumming hits (Zar, 2019). All statistical tests were conducted using R software (R Core Team, 2020).

## Results

### Tempo adjustment

**Figure 2:**
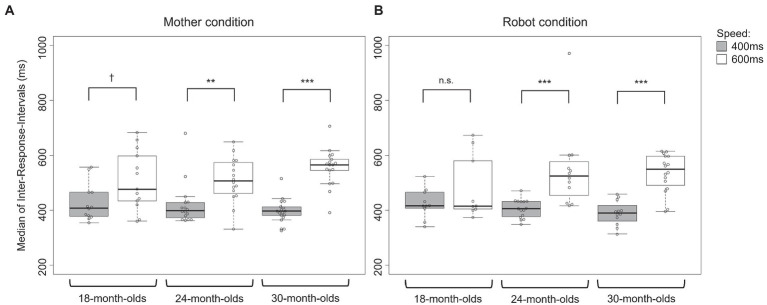
Median of inter-response intervals (IRIs) depending on the two speed conditions under **(A)**, mother condition and **(B)**, robot condition.

To test whether the children showed different drumming responses depending on the speed condition (400 or 600 ms ISI), we examined the median IRIs of each age group in the mother and robot conditions, respectively. In the mother condition (Figure 2A), the three age groups showed marginal or significant differences in drumming tempo between the 400 and 600 ms ISI conditions (Kruskal–Wallis rank sum test: 18-month-olds, *p* = 0.057; 24-month-olds, *p* < 0.01; 30-month-olds, *p* < 0.001). In contrast, in the robot condition (Figure 2B), while both the 24- and 30-month-olds showed a significant difference in drumming tempo depending on the speed condition (p < 0.001), no significant difference was observed in the 18-month-olds (*p* = 0.683). As shown in Supplementary Data S1, we further examined whether the variability of the drumming tempo or drumming frequency changed depending on the speed condition.

### SMT and tempo adjustment

**Figure 3:**
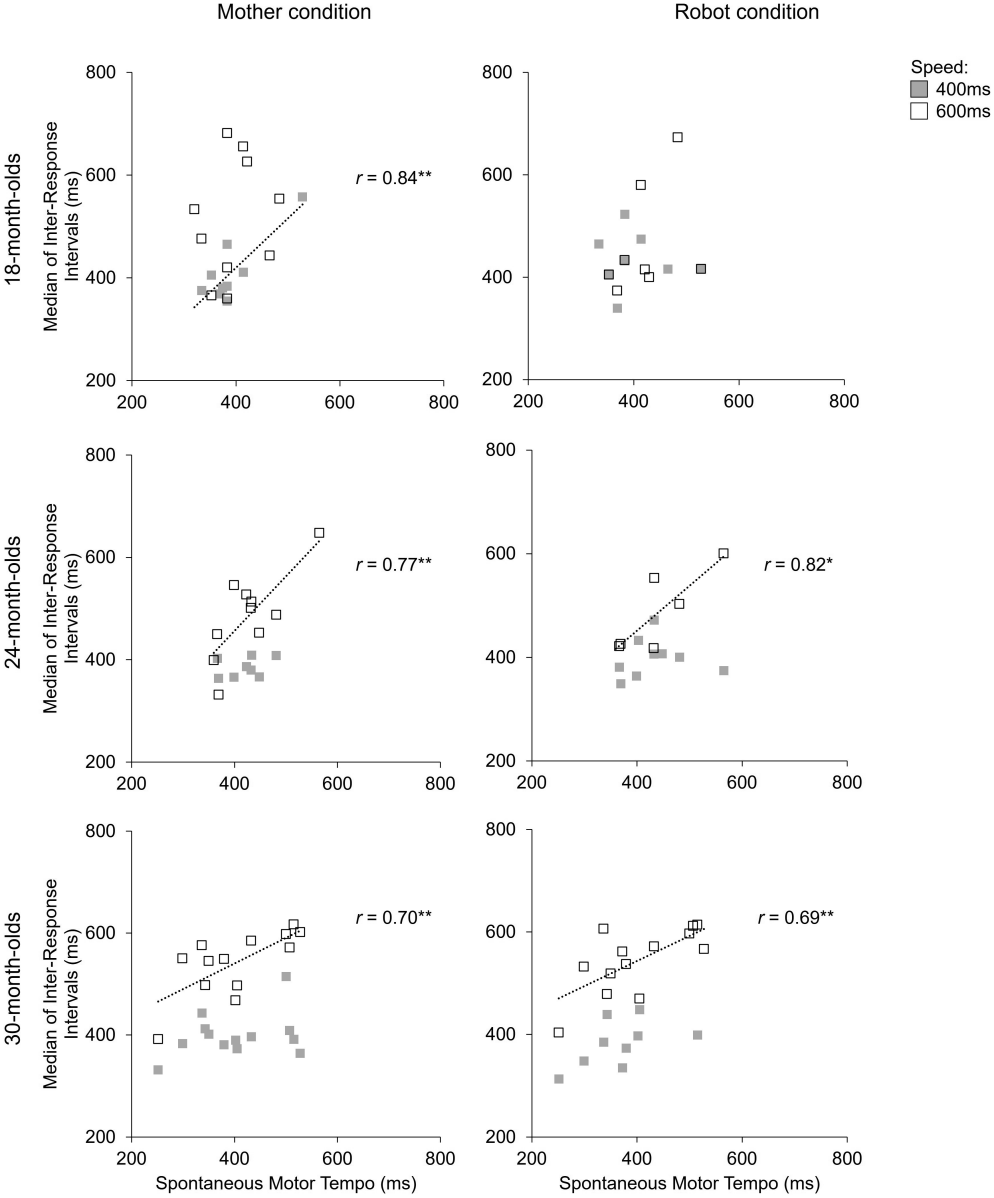
Correlation between the children’s spontaneous motor tempo (SMT) and the median of inter-response-intervals (IRIs) during the four test conditions.

To test whether the children who shifted to slower SMT were better at tempo adjustment, the relationship between the children’s SMT and the median IRI was examined in each condition (Figure 3). In the 24- and 30-month-olds, a positive correlation was found in the 600 ms ISI condition in both the mother and robot conditions (mother condition: 24-month-olds, *r*(8) = 0.77, *p* < 0.01; 30-month-olds, *r*(11) = 0.70, *p* < 0.01; robot condition: 24-month-olds, *r*(4) = 0.82, *p* < 0.05; 30-month-olds, *r*(11) = 0.69, *p* < 0.01). On the other hand, in the 18-month-olds, a positive correlation was found in the 400 ms ISI condition when they drummed in the mother condition. In Supplementary Data S2, we show the changes in the children’s SMT, the variability of the SMT, and the total number of drumming hits used for the SMT measurements across the three age groups. We found no significant correlation between SMT variability and tempo adjustment ability in any of the three age groups across the four test conditions.

### Synchronization and tempo adjustment

Table 1 shows the absolute number (*n*) and percentage (%) of children reaching significance in Rayleigh’s test (*p* < 0.05). The percentages were calculated as a ratio of the number of children who reached significance to the number of children tested. We found a developmental change in the absolute number of children who reached significance across the four conditions. In the 400 ms ISI condition, the number of children that reached significance tended to increase between the ages of 18 and 24 months. Conversely, in the 600 ms ISI condition, a prominent increase was observed between the ages of 24 and 30 months. These developmental trajectories were common in both the mother and robot conditions.

**Table 1:** Absolute number and percentage of children reaching significance in Rayleigh’s test.

Partner		Speed	18-month-olds			24-month-olds			30-month-olds		
		Tested	*n*	%	Tested	*n*	%	Tested	*n*	%
Mother	400 ms	8	3	37.5	15	7	46.67	17	8	42.06
	600 ms	7	3	42.86	16	3	18.75	17	11	64.71
Robot	400 ms	6	0	0	15	4	26.67	12	4	33.33
	600 ms	3	0	0	10	4	40	16	9	56.25

**Figure 4:**
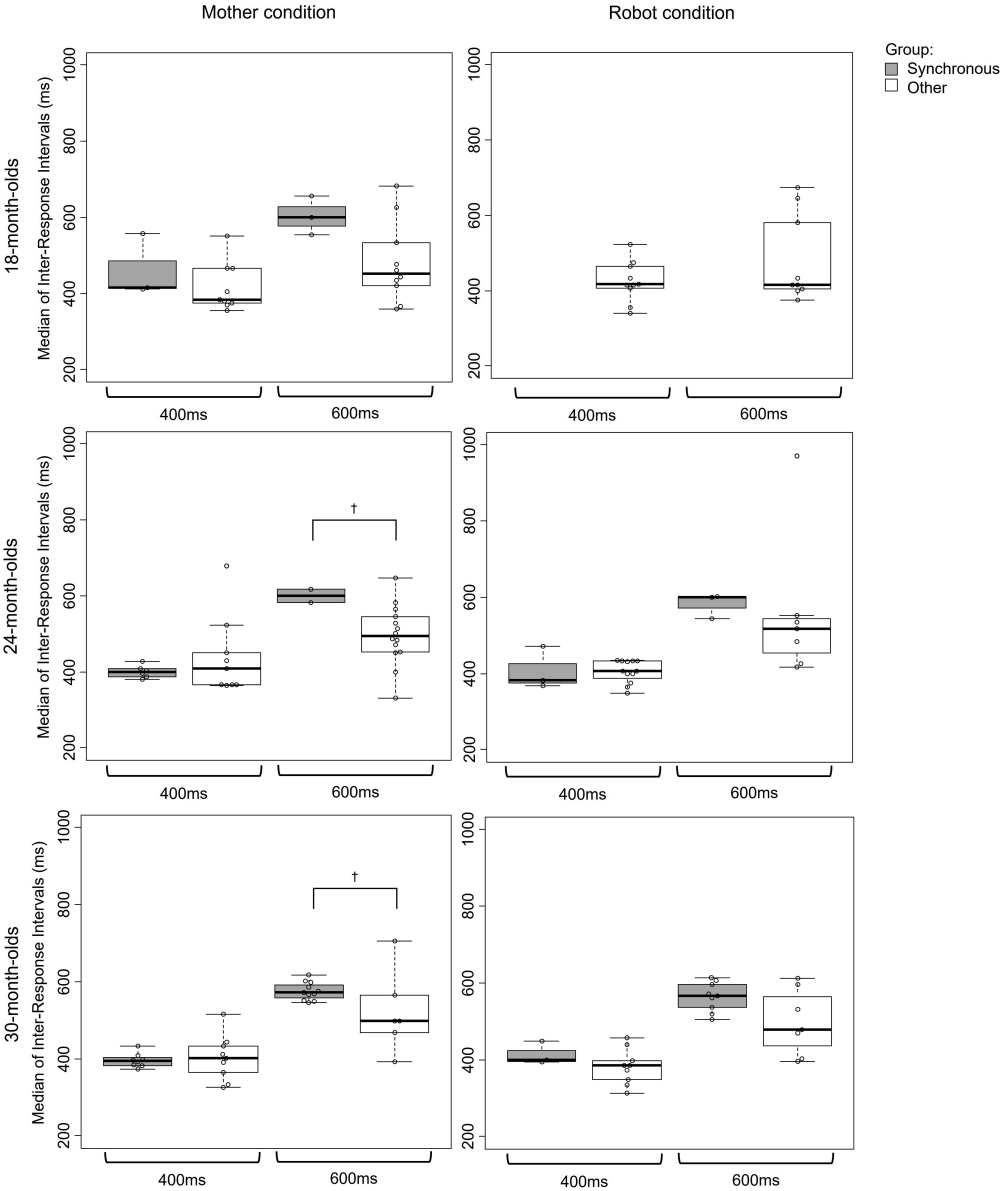
Relationship between the abilities of synchronization and tempo adjustment.

To examine the relationship between the abilities of accurate synchronization and flexible tempo adjustment, we first categorized the children into two groups: (1) the synchronous group, which included the children who reached significance in Rayleigh’s test and showed a phase preference between −90° and + 90° (i.e., in-phase synchrony); (2) the “other” group, which included the rest of the children. We then compared the tempo adjustment abilities between the two groups under the same speed condition. In both 24- and 30-month-olds, we found a marginal difference in the tempo adjustment ability between the synchronous and other groups in the 600 ms ISI condition when drumming in the mother condition (Kruskal–Wallis rank sum test: 24-month-olds, *p* = 0.068; 30-month-olds, *p* = 0.056; Figure 4).

## Discussion

The current study aimed to clarify the developmental process of flexible and accurate rhythmic coordination by examining when and how tempo adjustment and synchronization develop between 18-, 24-, and 30-month-old children. The most pivotal test condition in the current joint-drumming task was the 600 ms ISI speed condition. To coordinate with the 600 ms ISI, the children needed to slow down and control the timing of their drumming because the SMT for children around the ages of our participants was close to the 400 ms ISI (see also Provasi and Bobin-Bègue, 2003; Bobin-Bègue and Provasi, 2008; Rocha et al., 2021). The analysis of tempo adjustment indicated that children from the age of 24 months were able to slow down their drumming toward the 600 ms ISI condition, regardless of drumming partner. On the other hand, the analysis of synchronization indicated that children from the age of 30 months were able to control the timing with the onset of their partner’s drumming in the 600 ms ISI condition. These findings suggest that flexible tempo adjustment and accurate synchronization abilities develop gradually among 18-, 24-, and 30-month-olds.

The relationship between the children’s SMT and their tempo adjustment abilities demonstrated a significant correlation in 24- and 30-month-olds. This finding suggests that, as the children shifted to slower SMT (i.e., close to 600 ms), they were better at tempo adjustment during the 600 ms ISI condition. This further suggests that flexible tempo adjustments toward slower tempo than one’s own SMT develops later than tempo adjustments toward faster tempo in children. Regarding the positive correlation observed in the 18-month-olds, we assumed this result to be a false positive, as a child who showed an SMT close to 600 ms produced a drumming tempo of approximately 600 ms during the 400 ms ISI speed condition.

As previous studies have reported (e.g., Provasi and Bobin-Bègue, 2003; Bobin-Bègue and Provasi, 2008), the current study found that inter-individual differences in the children’s SMT tended to increase among the 18-, 24-, and 30-month-olds. This may be due to the divergence of the individual differences in the children’s motor development as their SMT shifts toward 600 ms, which is the SMT of adults (Fraisse, 1982; McAuley et al., 2006). Interesting further investigations may include clarifying the biological foundations of SMT, if an SMT of approximately 600 ms is a universal in adults of all cultures.

Although it was a marginal effect, the relationship between synchronization and tempo adjustment demonstrated that the children’s ability to synchronize accurately might have facilitated their tempo adjustment, especially in the 600 ms ISI condition. In the literature on human adults, two processes for error correction—period and phase correction—are known to act jointly to sustain accurate synchronization (Repp, 2005). Accordingly, during development, it is plausible that children use their ability to predict others’ actions to synchronize their own movements with those of another (i.e., phase correction), and this inevitably results in accurate tempo adjustments (i.e., period correction).

Compared to the two older age groups, children aged 18 months showed difficulty in flexible tempo adjustment and accurate synchronization toward the 600 ms ISI condition. Moreover, they showed few drumming hits across the four conditions. Our post-hoc video analysis indicated that most of the 18-month-olds exhibited rhythmic movements other than drumming behavior, such as head bobbing, body swaying, or bouncing (see Supplementary Data S3). As previous studies have reported (e.g., Zentner and Eerola, 2010; Fujii et al., 2014; Rocha and Mareschal, 2017; Cirelli and Trehub, 2019), it is plausible that the 18-month-olds were showing spontaneous rhythmic engagement with the music rather than attending to the partner to drum together. However, from the age of 24 months, the proportion of children showing other rhythmic movements decreased, and these children demonstrated more drumming hits compared to those of 18-month-olds. This suggests that the inhibition of other rhythmic movements, as well as attentional shift toward the partner (i.e., joint attention, see Siposova and Carpenter, 2019 for a review), were necessary for the children to produce drumming behavior in the current task.

In summary, we found that flexible tempo adjustment and accurate synchronization abilities develop gradually among 18-, 24-, and 30-month-olds in the joint-drumming task. Moreover, the findings demonstrated that both children’s motor development and cognitive ability to predict others’ actions function jointly in the development of flexible coordination in early childhood.

## Data availability statement

The data analyzed in this study are available from the corresponding author upon reasonable request.

## Ethics statement

The studies involving human participants were reviewed and approved by Kyoto University Unit for the Advanced Study of the Mind. Written informed consent to participate in this study was provided by the participants' legal guardian/next of kin.

## Author contributions

LY and MM designed the experiments and wrote the paper. LY and KT conducted the experiments. LY analyzed data. All authors contributed to the article and approved the submitted version.

## Funding

This study was financially supported by the JSPS Grants-in-Aid for Scientific Research (17H01016, 18H05524, 16F16001, 19K20646).

## Conflict of interest

The authors declare that the research was conducted in the absence of any commercial or financial relationships that could be construed as a potential conflict of interest.

## Publisher’s note

All claims expressed in this article are solely those of the authors and do not necessarily represent those of their affiliated organizations, or those of the publisher, the editors and the reviewers. Any product that may be evaluated in this article, or claim that may be made by its manufacturer, is not guaranteed or endorsed by the publisher.
