# Supplementary material for: From spontaneous rhythmic engagement to joint drumming: A gradual development of flexible coordination at approximately 24 months of age
Source: Front Psychol. 2022 Sep 29;13:907834. doi: 10.3389/fpsyg.2022.907834 (PMC9558294; doi:10.3389/fpsyg.2022.907834)
Supplement: Supplementary file 1 [file Data_Sheet_1.PDF]

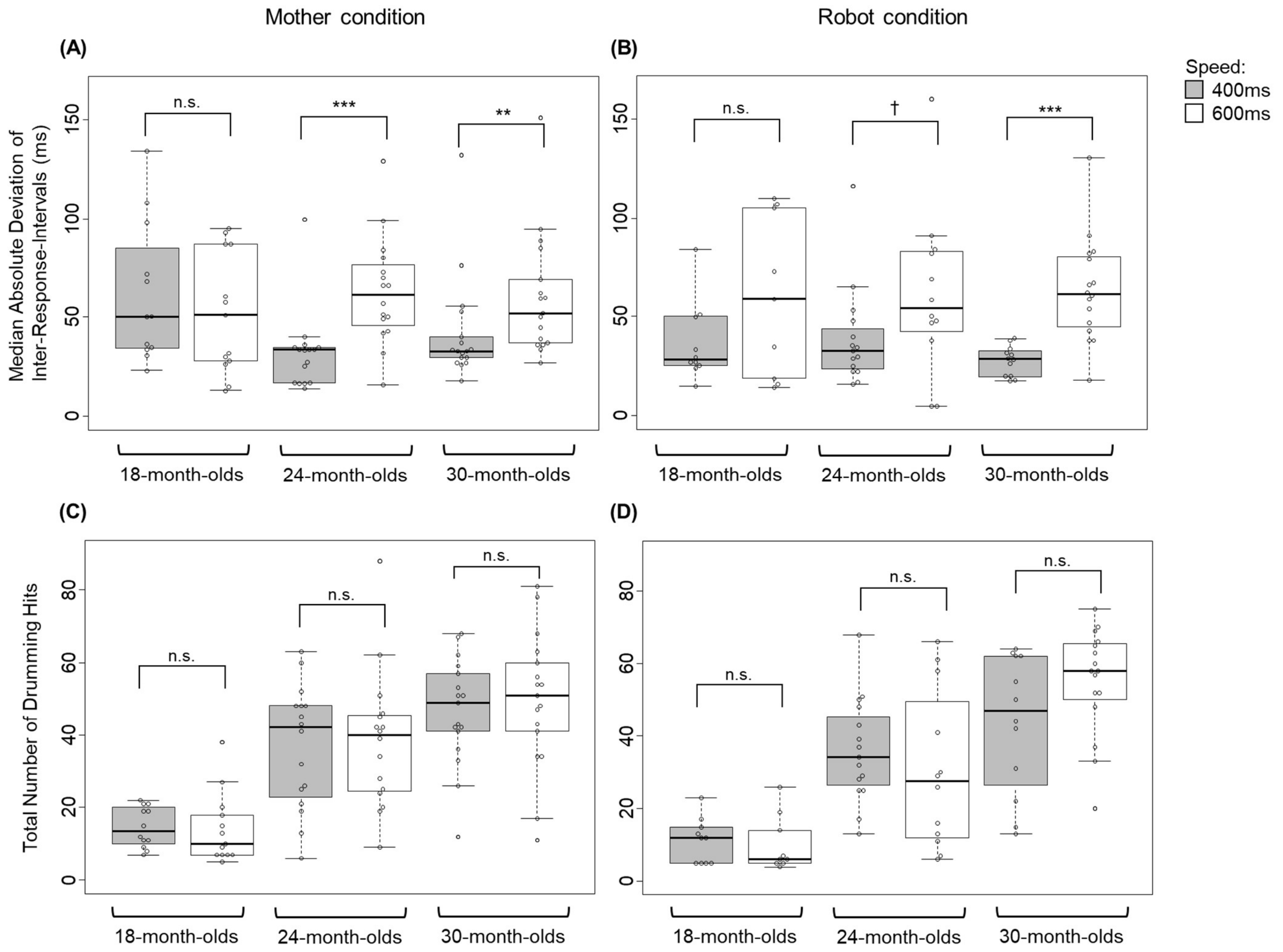

Supplementary Data S1. Tempo variability and total number of drumming hits.

To test whether the variability of the drumming tempo changed depending on the speed condition, we examined the median absolute deviation of the IRIs (S1(A) and (B)). In both the mother and robot conditions, 18-month-olds showed no significant differences in tempo variability depending on the speed condition (mother condition, $p = .384$; robot condition, $p = .327$), while the 24- and 30-month-olds showed marginal or significant differences (mother condition: 24-month-olds, $p < .001$; 30-month-olds, $p < .01$; robot condition: 24-month-olds, $p = .06$; 30-month-olds, $p < .001$). Next, to test whether the children's drumming frequency changed depending on the speed condition, we examined the total number of drumming hits (S1(C) and (D)). All three age groups showed no significant differences in both the mother and robot conditions (mother condition: 18-month-olds, $p = .325$; 24-month-olds, $p = .821$; 30-month-olds, $p = .581$; robot condition: 18-month-olds, $p = .803$; 24-month-olds, $p = .305$; 30-month-olds, $p = .09$).

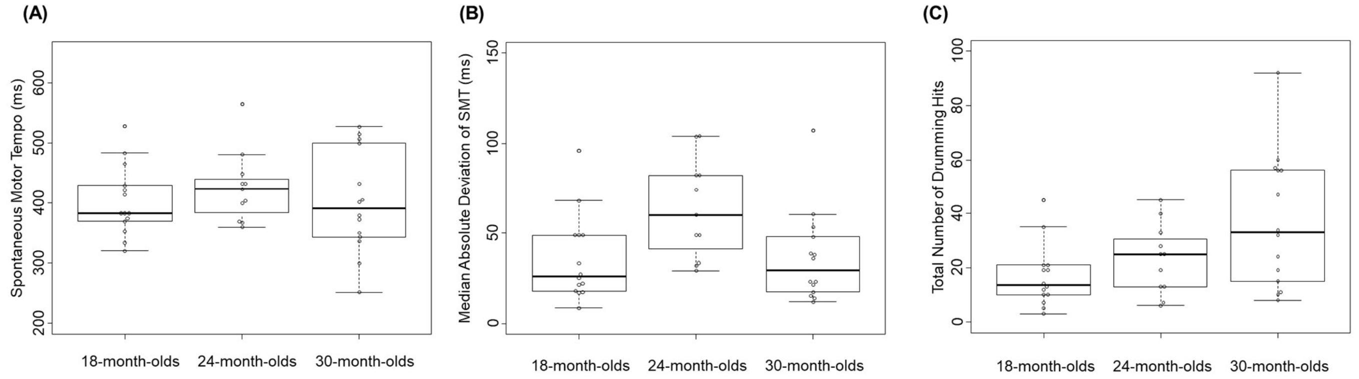

Supplementary Data S2. The children's spontaneous motor tempo (SMT), the SMT variability, and the total number of drumming hits that were used for the SMT measurements across the three age groups.

The SMT was extracted from each participant's drumming responses during the familiarization phase. We found that the median SMT was not significantly different among the three age groups (Kruskal–Wallis rank sum test: $p = .595$; S1(A)), reaching approximately 400 ms. Regarding the variability of the SMT, we found a significant age effect ($p < .05$; S2(B)). The post-hoc tests revealed that the 24-month-olds showed a significantly higher variability than the 18-month-olds (pairwise Mann–Whitney U tests with Bonferroni correction: $p < .05$), while there was a marginal difference between the 24- and 30-month-olds ($p = .056$). No significant difference was found between the 18- and 30-month-olds. Regarding the total number of drumming hits, we found a significant age effect ($p < .05$; S2(C)). The post-hoc tests revealed a marginal difference between the 18- and 30-month-olds ($p = .057$), while there was no significant difference between the 18- and 24-month-olds and between the 24- and 30-month-olds.

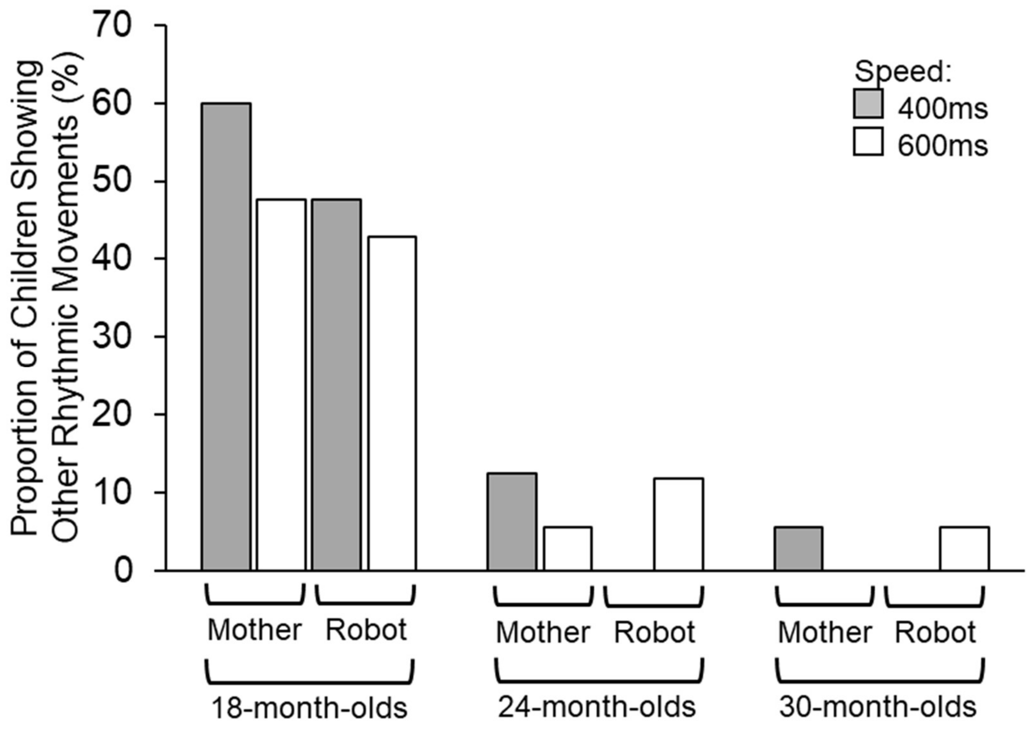

Supplementary Data S3. Proportion of children showing other rhythmic movements depending on each condition.

Rhythmic movements other than drumming behaviors were identified using the ELAN software and were defined as when the children showed two or more cycles of rhythmic movements (i.e., head bobbing, bouncing, and so on) within 2 seconds. If children demonstrated at least one type of rhythmic movement, we counted the proportion of children showing other rhythmic movements.
